# Too hip for two sacral vertebrae

**DOI:** 10.7554/eLife.53399

**Published:** 2019-12-19

**Authors:** Michelle R Stocker

**Affiliations:** Department of GeosciencesVirginia TechBlacksburgUnited States

**Keywords:** Crocodylia, variation, Hox, Miocene, evo-devo, gigantism, Other

## Abstract

A complex pelvic morphology has been discovered in the fossils of one of the largest crocodylians.

**Related research article** Scheyer TM, Hutchinson JR, Strauss O, Delfino M, Carrillo-Briceño JD, Sánchez R, Sánchez-Villagra MR. 2019. Giant extinct caiman breaks constraint on the axial skeleton of extant crocodylians. *eLife* **8**:e49972. doi: 10.7554/eLife.49972

The properties of vertebrae – the bones that make up the backbone – have a crucial influence on the way that mammals, birds, fishes and many other vertebrate species move. Some vertebrates have vertebrae that are rather charismatic in terms of size, shape or number. For example, all mammals possess seven vertebrae within their necks, whether they are a giraffe or a mouse, and the length of these vertebrae depends on the length of the neck.

Reptiles typically have two vertebrae in their sacrum, which is near the pelvis (Hoffstetter and Gasc, 1969). Having two sacral vertebrae is characteristic of the Archosauromorpha, a group that includes crocodylians (large, predatory, aquatic and semiaquatic reptiles) and their extinct close relatives (Nesbitt, 2011; Griffin et al., 2017). However, birds and their close relatives among non-avian theropods (dinosaurs with hollow bones and three toes) deviate from this trend of having two vertebrae in the sacrum. Many birds have at least 12 sacral vertebrae, and swans have 24 (Turner et al., 2012)!

Studies of growth in living species, like the chicken, show that the ordering of the body during development – and the number of vertebrae formed – is controlled by a group of genes called the *Hox* genes. These genes have recently been shown to control patterning in other archosauromorphs, including crocodylians (e.g. Böhmer et al., 2015).

But this is all in living species, what about fossils? It is known, for example, that some early theropod fossils (like those of *Tyrannosaurus* or *Velociraptor*) exhibit more than two sacral vertebrae (Turner et al., 2012). Exploring the vertebrae of fossils helps to fill in the gaps in our understanding of morphology, and helps explain why modern groups look the way they do and how extinct members of those groups organized their body plans.

Now, in eLife, Torsten Scheyer of the University of Zurich and co-workers in Switzerland, the United Kingdom, Italy, Spain and Venezuela report on a crocodylian fossil that has more than two sacral vertebrae (Scheyer et al., 2019). The extinct *Purussaurus mirandai*, one of the largest known crocodylians, is found fossilized in rocks in northern South America that are between 5 and 13 million years old, and is related to crocodylians alive today. This giant caiman is known for its very large skull, but Scheyer et al. have now described all the other bones known to be associated with the species. As part of that description, they examined the sacral vertebrae and found the first non-pathological case of a crocodylian with more than two vertebrae in their sacrum (Figure 1).

**Figure 1.**
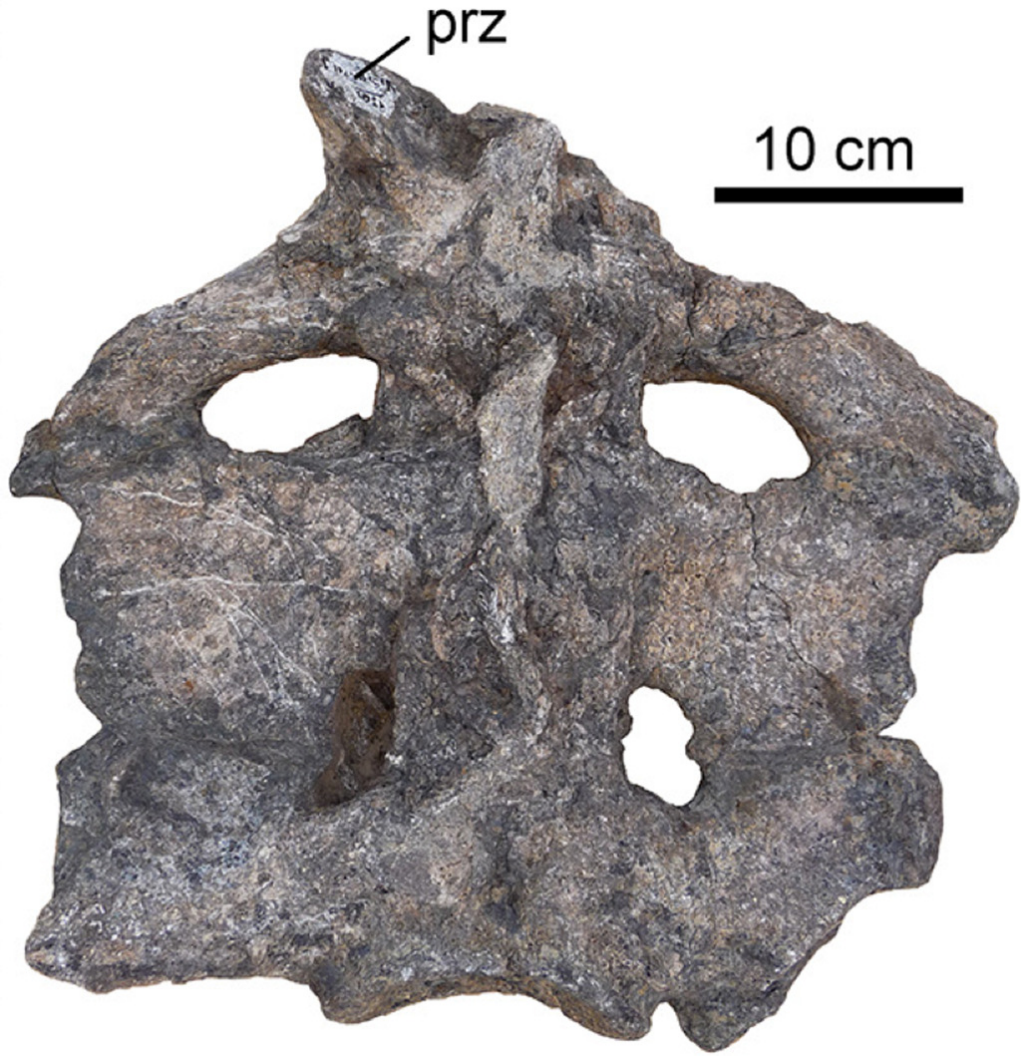
Fossilized sacral vertebrae of *Purussaurus mirandai*. Scheyer et al. have examined the skeleton of the crocodylian *P. mirandai*. All other crocodylians examined to date only have two vertebrae in their sacrum, making *P. mirandai* the first reported case of a crocodylian with three sacral vertebrae. This is likely due to a change in the expression of the *Hox* genes in this species. Indicated as prz in the image, the prezygagophysis connects each vertebra with the anterior one in the spine.

How did the number of vertebrae increase? As the skeleton grows, three types of changes are possible: a vertebra could be added from the tail (i.e., the ‘caudal’ vertebrae), creating a caudosacral; a vertebra could be inserted between the original two; or a vertebra could be added from the front, creating a dorsosacral. Scheyer et al. show that *P. mirandai* has the two original sacral vertebrae as well as a dorsosacral, similar to some extinct crocodylian-like animals called phytosaurs (Griffin et al., 2017).

But why would a species add vertebrae? An expanded sacrum might give vertebrates increased stability. This is important when a species increases in size, or when it becomes bipedal (and has to be able to balance while standing on two legs). Scheyer et al. looked at other parts of the skeleton to answer why *P. mirandai* had an extra vertebra, examining the shoulder girdle. In these specimens, the shoulder girdle is oriented more vertically than in other crocodylians with just two sacral vertebrae. This, along with the expanded pelvis, is evidence for weight being supported by the limbs rather than the trunk, hinting at the species becoming more upright. Crocodylians also have massive, muscular tails, which add to the weight that has to be supported and also changes the center of mass (Molnar et al., 2014).

These newly examined specimens of *P. mirandai* show that crocodylians have the ability to expand the number of sacral vertebrae, suggesting a change in the pattern of *Hox* gene expression in this species. There is evidence that other non-dinosaurian archosauromorphs, such as phytosaurs, expanded their sacral vertebrae, too, despite having evolved separately (Griffin et al., 2017). However, a sacrum with more than two sacral vertebrae has evolved multiple times independently, especially in animals from the Triassic Period (~250–200 million years ago). The confirmation that crocodylians can have more than two sacral vertebrae rewrites what was thought of as possible for this group of animals, adding an interesting new layer of developmental and morphological flexibility.
